# Poor awareness and attitudes to sanitation servicing can impede China's Rural Toilet Revolution: Evidence from Western China

**DOI:** 10.1016/j.scitotenv.2021.148660

**Published:** 2021-11-10

**Authors:** Shaomin Guo, Xiaoqin Zhou, Prithvi Simha, Luis Fernando Perez Mercado, Yaping Lv, Zifu Li

**Affiliations:** a Beijing Key Laboratory of Resource-oriented Treatment of Industrial Pollutants, School of Energy and Environmental Engineering, University of Science and Technology Beijing, Beijing 100083, PR China; b Department of Energy and Technology, Swedish University of Agricultural Sciences, Box 7032, SE-750 07 Uppsala, Sweden; c Center for Water and Environmental Sanitation (Centro de Aguas y Saneamiento Ambiental, CASA), Universidad Mayor de San Simon, Calle Sucre y Parque Latorre, Cochabamba, Bolivia

**Keywords:** Rural sanitation, Nutrient recycling, Pit latrines, Social norms, Source separation, Wastewater treatment

## Abstract

The ongoing Toilet Revolution in China offers an opportunity to improve sanitation in rural areas by introducing new approaches, such as urine source separation, that can contribute to achieving SDG6. However, few studies have systematically assessed the social acceptability of managing human excreta collected in new sanitation systems. Therefore, in this study we performed face-to-face interviews with 414 local residents from 13 villages across three provinces in western China, to analyze the current situation and attitudes to possible changes in the rural sanitation service chain. We found that the sanitation chain was predominantly pit latrine-based, with 86.2% of households surveyed collecting their excreta in a simple pit, 82% manually emptying their pits, and 80.2% reusing excreta in agriculture without adequate pre-treatment. A majority (72%) of the households had a generally positive attitude to production of human excreta-derived fertilizer, but only 24% agreed that urine and feces should be collected separately. Multivariate logistic regression indicated that three factors (level of education, number of permanent household residents, perceived social acceptability) significantly influenced respondents' attitudes to reuse of excreta, although only perceived social acceptability had a high strength of association. Overall, our survey revealed that rural households often misuse toilet systems, fail to comply with government-specified sanitation guidelines, have low awareness of alternative solutions, and are over-reliant on the government to fix problems in the service chain. Thus while new sanitation technologies should be developed and implemented, information campaigns that encourage rural households to manage their excreta safely are also important.

## Highlights

• Face-to-face interviews were performed at 414 rural households in western China.
• Both status and attitude towards the rural sanitation service chain were evaluated.
• Poor hygiene practices are ubiquitous across the service chain, posing health risk.
• Perceived social acceptability correlated with positive attitude to excreta reuse.
• Awareness campaigns can positively influence sanitation management.

## Graphical abstract

**Figure:**
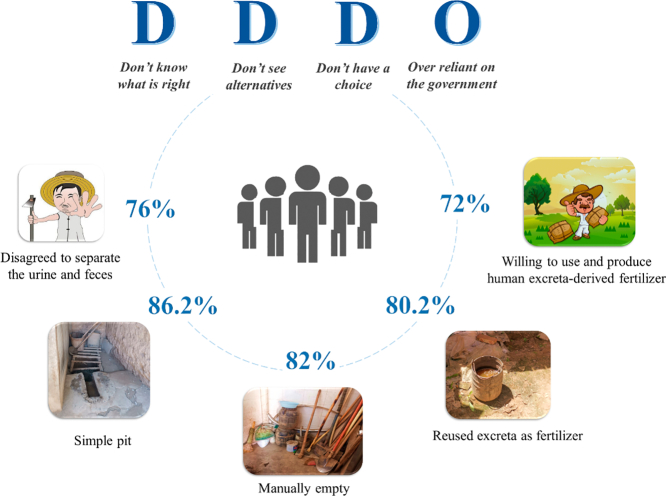

## Nomenclature

MOHC: Ministry of Health of the People's Republic of China
SDG6: United Nations Sustainable Development Goal 6: ‘Ensure availability and sustainable management of water and sanitation for all’
SARS: Severe Acute Respiratory Syndrome

## 1 Introduction

With the launch of the Toilet Revolution program in China in 2016, increasing attention has been paid to sustainable sanitation in rural areas, with different degrees of progress being made in different regions (Cheng et al., 2018). In 2012, the Ministry of Health of the People's Republic of China (MOHC) released guidelines that officially recommend six types of sanitary toilets (three-septic-tank, double-vault funnel, biogas-linked, integrated flushing, urine–feces division, and double-pit alternate toilets) for rural areas (GB19379-2012). The guidelines also provide detailed specifications on toilet design, construction, and excreta management. The sanitary toilets recommended by MOHC have increased in frequency, from 44.8% in 2000 to 81.7% in 2017 (National Bureau Of Statistics, 2020). However, this means that around 18.3% of rural households (about 48 million households) still do not have their own sanitary toilet. In other words, transforming rural sanitation in China and improving the state of its toilets and human excreta management still remains a considerable challenge.

Conventional on-site sanitation systems, including dry systems (pit toilets) or water-based systems (flush toilets) for managing excreta at household level still dominate in rural areas in developing countries (Dasgupta et al., 2020). In 2017, around 2.7 billion (38%) people world-wide used on-site sanitation facilities, including pit latrines and septic tanks, because these are inexpensive and have low infrastructure requirements (Koné, 2010). In such systems, having a safe sanitation service chain is essential, because excreta must be hygienically emptied, transported, and adequately treated for reuse or disposal when toilets are full (Capone et al., 2020; van Welie et al., 2019). China has a long history of using human excreta in agriculture and farmers generally view latrine waste as “valuable fertilizer” (Ferguson, 2014). Although this practice is traditional and has been followed over generations, it poses human health risks. If excreta are inadequately treated, any pathogens present during application in agriculture can enter soils, groundwater, and the food chain, thereby threatening human health (Phuc et al., 2006).

For on-site sanitation systems to be sustainable, the service chain must be technically feasible and acceptable to users (Simha and Ganesapillai, 2017; Werner et al., 2009). Social acceptance is widely believed to be a major barrier to implementing on-site systems, such as those based on urine diversion. According to a survey in Madagascar (Moya et al., 2019), the main barrier to using human excreta-derived fertilizers is lack of awareness of new products, not moral prejudice. A survey in the USA found that respondents believed that human excreta did not differ from biosolids and that perceived benefits were the main factor determining reuse, i.e., if people understand the value of excreta-based products, then acceptance is high (Segrè Cohen et al., 2020). In a study in India, 59% of farmers surveyed were positive to reuse of urine and 46% were positive to reuse of human feces (Simha et al., 2017). However, farmers in that study were more positive to their neighbors, rather than their friends, family, and colleagues, applying human urine, a behavior that the authors called “not in my circle” (Simha et al., 2017). A study in Ethiopia found that household size, farm income, and frequency of organic fertilizer application negatively influenced the decision to use human excreta-derived fertilizers, while number of livestock and farm size had a positive effect (Gelgo et al., 2016). However, in a survey in China, farm size had a negative effect on the amount of human excreta-derived fertilizers used, while planting years had a positive effect and education level of the interviewee had no significant effect (Wang et al., 2018). Therefore, the factors that significantly affect use of human excreta-derived fertilizers appear to vary in different regions. This is confirmed by a recent global survey of 3763 people in 16 countries, which found that consumer willingness to eat food fertilized with human urine differs greatly by country and that the strongest predictive factors for overall acceptance are cognitive factors (perceptions of risks and benefits) and social norms (Simha et al., 2020).

In China, few studies to date have examined the status of the sanitation service chain, especially in rural areas, and limited research has been performed on rural residents' perceptions of human excreta-derived fertilizers and source separation. Therefore in the present study, we surveyed 13 villages in three provinces in western China, with the objectives of (i) exploring the status of the sanitation service chain in rural areas in China, (ii) identifying factors that affect people's attitudes to production and use of human excreta-derived fertilizers in agriculture, and (iii) identifying factors that affect the development of source-separating sanitation systems. The overall aim was to improve understanding of social issues affecting implementation of new sanitation systems that promote nutrient recycling. Such knowledge could help guide policymakers and decision-makers in effectively promoting China's Toilet Revolution. It also could act as reference for promoting new sanitation practices that enhance the availability and sustainability of water and sanitation services in other low-middle income countries worldwide, ultimately contributing to achieving SDG6.

## 2 Methodology

### 2.1 Survey

The survey was conducted between April 20 and July 12, 2019, in 13 villages located in three provinces (Gansu, Sichuan, and Qinghai) in western China. Face-to-face interviews were performed by nine Chinese researchers majoring in environmental engineering, and the responses of the interviewees were recorded in a pre-designed questionnaire. The villages were chosen on the basis of local government recommendations and were known to have high usage of dry toilets/latrines and potential for producing organic fertilizers. The sanitation chain covered by the survey included user interface, storage, conveyance, treatment, and use or safe disposal of human excreta. The households were selected through simple random sampling of the local residents' register. Before the start of the interview, the interviewees were informed about the purpose of the survey, that their participation was voluntary, and that responses would be recorded anonymously.

The questionnaire was designed based on previous literature. To validate the questions, determine correct expressions, and eliminate ambiguity and redundancy, a test survey was performed with an expert group of eight research scholars in related fields. A two-way preliminary test was also conducted on the questionnaire, to check the appropriateness and clarity of the content, and 10 volunteers from rural households were selected for a pre-survey. The final questionnaire was prepared and completed after incorporating the recommendations of the expert group and the volunteers. The preliminary survey revealed that, due to differences in the language and living habits of residents in different rural areas in China, the survey questions should be simple and easy to understand and the interviewers should explain the questionnaire in plain language. A concise questionnaire was finally prepared, and the nine interviewers were trained in local dialects and survey methods.

The questionnaire had a series of 17 sequential closed-ended questions with multiple choice answers and was divided into three main sections (see Supplementary information). In Section A (questions Q1–Q6), the socioeconomic profile of the interviewees was established; in Section B (Q7–Q10), details of the current sanitation service chain were obtained; and in Section C (Q11–Q17), the interviewees' attitudes to reuse of human excreta were determined. In Sections A and B, single responses to closed-ended questions that were either binary (yes/no) or multiple-choice type were obtained. In Section C, some logical questions were included or multiple responses were provided (i.e., more than one answer could be chosen).

### 2.2 Data processing and analysis

The survey data were statistically analyzed to identify factors affecting the willingness of the respondents to produce fertilizers from human excreta. Logistic regressions were performed using the questions from Sections A and B (1–6 and 7–10) as independent variables and question 11 from Section C (Would you like to produce fertilizers derived from human excreta?) as the dependent variable. In order to enable logistic regression on the data, binary questions were assigned a value of 0 and 1 for negative and positive answers, respectively. The questions with ordinal categories were converted to numerical answers by assigning them integer numbers. Lastly, multiple-choice questions were treated as categorical variables, although some multiple-choice questions were converted to a binary response when appropriate. For example, question 4 had six choices for occupation, but the answers were transformed to a binary variable (agricultural occupation yes/no), as we expected high willingness to produce human-excreta fertilizers among agricultural workers. The logistic regression models tested were composed of: a) all the independent variables as predictors and b) each independent variable as a predictor. McFadden pseudo R^2^ was used to compare the models, and R statistical software was used for statistical analysis. The coding applied is shown in Supporting information Part II.

## 3 Basic information on interviewees

Full sociodemographic information on the interviewees is presented in Table 1. The proportion of male interviewees was slightly higher than that of female interviewees, possibly because in Chinese culture males act as family representatives and take the lead in communicating with guests, especially in rural areas. Males are also the main labor force in sanitation and more familiar with the management of toilets and toilet wastes. The majority of the interviewees were aged between 40 and 60 years, which correlated well with the overall rural village population, as young people migrate to urban areas (Cao et al., 2014). The interviewees mostly had primary or junior high education level and their main occupation was farming. About 38% of the households had net annual income below CNY10,000. In 2018, the average rural per capita disposable income in western China was CNY11,831 (National Bureau Of Statistics, 2019). Thus, the majority of households were at low or middle-income level. However, most of the interviewees were unwilling to disclose their real income, so the data are only a reference value and may not represent the real situation. The permanent household residents surveyed were usually two people, although this number increased from two to 10 people during holiday periods.

**Table 1:** Sociodemographic characteristics of the interviewees.

Variable	N	Proportion
*Gender*		
Female	193	46.8%
Male	221	53.2%

*Age (years)*		
≤30	42	10.4%
31–40	51	12.1%
41–50	113	27%
51–60	122	30%
≥60	86	20.6%

*Education level*		
None	98	23.4%
Primary school	118	28.1%
Junior high school	128	31.2%
High school	44	10.4%
College	16	4.3%
Bachelor degree or above	10	2.6%

*Occupation*		
Unemployed	69	17.3%
Farmer	271	64.1%
Worker	45	10.9%
Businessman	9	2.6%
Students	9	2.6%
Other	11	2.6%

*Annual net income (CNY)*		
≤10,000 yuan	158	37.4%
10,000–20,000 yuan	96	22.9%
20,000–40,000 yuan	96	22.7%
40,000–60,000 yuan	24	5.9%
≥60,000 yuan	9	3.8%
Unknown	31	7.3%

*Number of permanent household residents*		
1	17	4.3%
2	117	27.9%
3	69	16.3%
4	66	16.1%
5	65	15.8%
6	50	11.8%
≥7	30	7.8%

## 4 Results and discussion

### 4.1 Current status of the sanitation service chain

To characterize the current status of the sanitation service chain, we collected data on five components: user interface, storage, conveyance, treatment, and use or safe disposal of waste. The results showed that dry toilets were the dominant type of toilets used by the respondents (84%) (Table 2). Only 15% had access to flush toilets, including pour flush and cistern flush toilets. About 1% of the interviewees used a public or shared toilet because they did not have their own toilet.

**Table 2:** Current status of the sanitation service chain in the rural area surveyed.

	User interface			Storage			Conveyance			Treatment and reuse		
	Type	N	Proportion	Type	N	Proportion	Type	N	Proportion	Type	N	Proportion
A	Dry latrine	348	84.1%	Simple pit toilet	357	86.2%	Manual emptying	340	82.1%	Reuse as crop fertilizer	332	80.2%
B	Flushing toilet	62	15.0%	Biogas-linked toilet	22	5.3%	Suction truck	62	15.0%	Unknown	59	14.3%
C	Public or shared toilet	4	0.1%	Three-septic-tank type	18	4.4%	Sewage pipe	11	2.7%	Dumped in environment	12	2.9%
D	Other type	0	0%	Integrated flushing toilet	11	2.7%	Unknown	1	0.2%	Wastewater treatment plants	11	2.7%
E				Public or shared toilet	4	0.1%						
F				Urine–feces division toilet	2	0.5%						
G				Double pit alternate type	0	0%						
H				Double-vault funnel type	0	0%						
**Total**		**414**	**100%**		**414**	**100%**		**414**	**100%**		**414**	**100%**

**Fig. 1:**
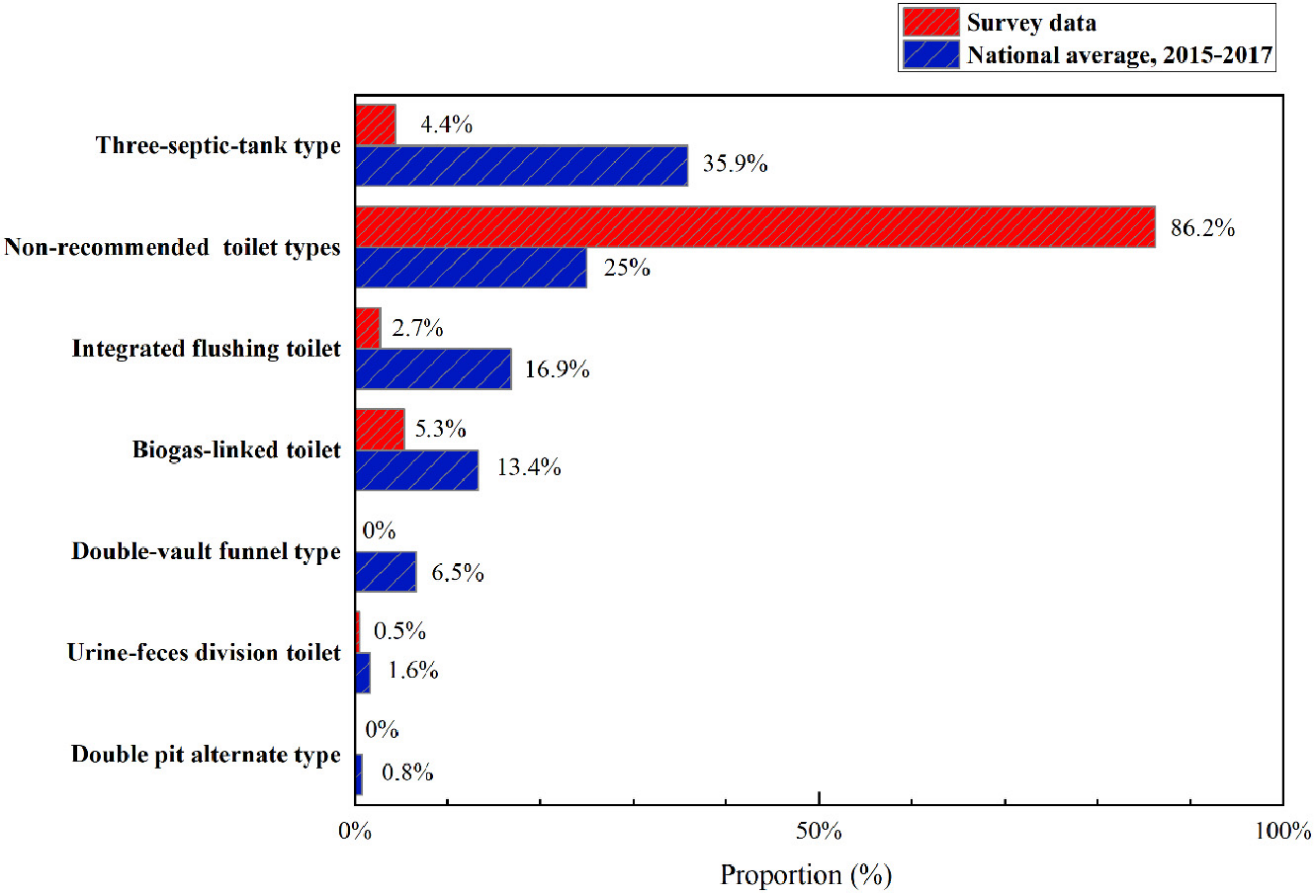
Proportion of rural households surveyed using different types of toilets and the national average 2015–2017.

We found that 86% of our survey respondents collected and stored their excreta in a simple pit and did not use the officially recommended toilets. Use of all six toilet types recommended by the Chinese MOHC guidelines (GB19379-2012) was lower among respondents than the official average in 2015–2017 (National Bureau Of Statistics, 2020) (Fig. 1). Three-septic-tank type toilets, integrated flushing toilets, and biogas-linked toilets were rarely seen in the survey areas, and almost all the biogas-linked toilets were in Sichuan (Table 3). However, many respondents reported that they only used their biogas-linked toilets as simple pits, because of low biogas production and inconvenient operation. In other words, most of biogas-linked toilets were used as simple pit latrines, and the mixture of feces and urine captured in simple pits/containers was simply stored rather than treated. In many Chinese provinces, three-septic-tank type toilets are widely recommended for reforming rural sanitation, because of their low construction costs, short excreta storage times, and simpler use than other toilet types (Bin, 2020). However, these toilets accounted for only 4.4% of all toilets used by the rural residents we surveyed. Although 15% of our interviewees used flush toilets, only 2.7% of them had integrated flush toilets connected to a sewage system. The majority were either connected to three-septic-tank types, simple pits, or biogas reactors, because of incomplete sewage infrastructure in most villages. Double-pit alternate, double-vault funnel, and urine-feces division toilet types were used by very low proportions (<1%) of the respondents (Fig. 1).

**Table 3:** Reponses from in the three provinces to Question 8 of the survey.

		Q8. How do you store your family excreta?						Total
		A. Three-septic-tank type	C. Biogas-linked toilet	D. Urine–feces division toilet	E. Integrated flushing toilet	G. Simple pit	H. Public or shared toilet	
Province	Gansu	1	1	1	2	305	4	314
	Qinghai	0	0	1	2	41	0	44
	Sichuan	17	21	0	7	11	0	56
Total		18	22	2	11	357	4	414

Manual self-emptying of toilet waste was the most common mode of emptying, practiced by more than 82% of the respondents, while about 15% of the households paid a worker to empty their human excreta, at an average cost per occasion of 80 to 120 Yuan. Less than 2% of respondents' toilets (1.6% in Sichuan, 0.4% in Qinghai, 0.4% in Gansu) emptied directly into sewer pipes, which only existed in villages with sufficient water supply and sewage infrastructure.

In terms of treatment and reuse, 80% of the collected excreta was returned to agriculture and reused as a crop fertilizer. According to the Chinese MOHC standards GB7959-2012, GB19379-2012, and GB/T38837-2020, human excreta should be applied as a fertilizer in agriculture only after at least two months of storage in a standard three-septic-tank/double-vault funnel-type toilet or after at least six months to one year of sealed storage in other toilet types (e.g., biogas-linked, double pit alternate, urine-feces division). However, during our site visits, we observed that fresh and stale excreta were routinely mixed in simple pits and biogas reactors by 91.5% of the households surveyed. Fresh excreta were continuously added before the next emptying, indicating that the collected excreta were not treated hygienically, irrespective of the interval between emptying schedules. Most of the three-septic-tank types were constructed privately and it is difficult to say whether they were designed and operated properly, and whether there were risks to human health. Prior research indicates a high concentration of pathogens in sludge from septic tanks (Foster et al., 2021; Peal et al., 2020; Amin et al., 2020; Mills et al., 2018). We found cases in which the third compartment was very large, so that the ratio of the three compartments was not 2:1:3, to reduce the frequency of emptying. Moreover, in some cases three-septic-tank systems were smaller than the recommended size because of space limitations and tanks had to be emptied frequently. About 2.9% of the human excreta collected in simple pits (1.93%), biogas-linked toilets (0.24%), and three-septic-tank toilets (0.72%) was discharged without any treatment. In Sichuan, 1.2% was found to be seeped into the ground, while 1.4% and 0.2% was directly dumped in the environment in Gansu and Qinghai, respectively. Therefore, only the roughly 2.7% of excreta sent to wastewater treatment plants were treated hygienically, and almost all excreta from the surveyed households were actually dumped on farmland without proper treatment.

**Fig. 2:**
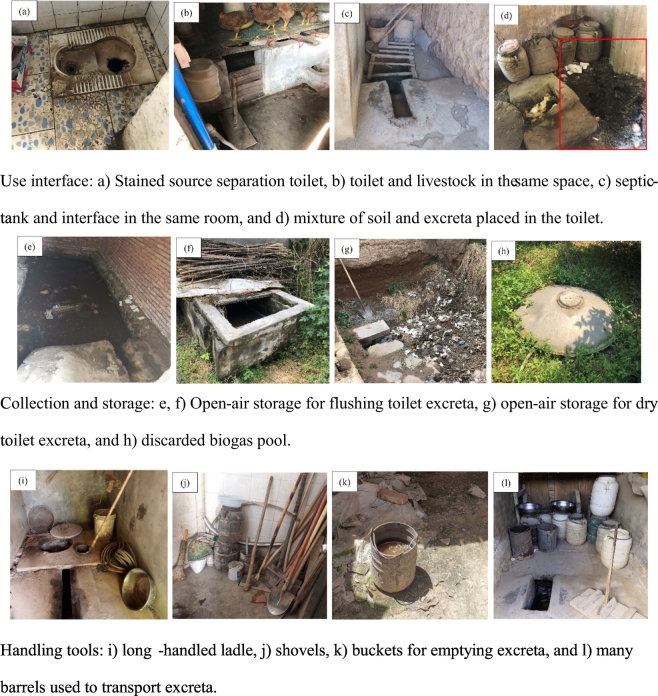
Pictures depicting the poor state of the sanitation chain in the survey area (images taken by the interviewers).

Based on our results, the rural sanitation service chain in Sichuan, Qinghai, and Gansu provinces is clearly not in line with the requirements for sustainable management of human excreta. The main service chain is pit latrine-based and there are multiple problems associated with poor hygiene. Examples in Fig. 2 show a stained source separation toilet because of insufficient flushing water (Fig. 2a), toilets with unsuitable structures (Fig. 2b–d); open-air storage of excreta (Fig. 2b–g), and crude tools for excreta emptying (Fig. 2i–l). All these shortcomings can increase the spread of contaminants (Ignacio et al., 2018).

In China, over-application of fertilizers at above the optimal rate is common and farmers prefer nitrogen and compound fertilizers (Zheng et al., 2020). For instance, an average of about 2715 kg/ha of chemical fertilizer is applied by farmers in Shaanxi province, while human excreta-derived fertilizer is preferred by less than 3% of farmers (Wang et al., 2018). According to Chinese statistics (National Bureau Of Statistics, 2019), chemical fertilizer consumption in Sichuan, Gansu, and Qinghai in 2019 was 802.05, 622.06, and 387.85 kg/ha/year, respectively (Table 4). These values are, respectively, 3.5, 2.7, and 1.7 times the international upper limit of 225 kg/ha for safe application of chemical fertilizers (Zheng et al., 2020). Although no reliable data are available on the use of human excreta and organic fertilizer in these three provinces, we do not expect the usage to be any different from that reported recently for Shaanxi province (Wang et al., 2018). It has been predicted that, through manure recycling, by 2050 China's demand for synthetic N fertilizer could be reduced by about one-third (about 10 Mt), which is equivalent to 10% of current global fertilizer N consumption (Zhang et al., 2020). However, achieving this will be difficult because of farmers' negative attitudes to manure use (Zhang et al., 2020). The high use of chemical fertilizers and inadequate utilization of human excreta-derived fertilizers indicate that safe use of human excreta should be maximized.

**Table 4:** Amount of fertilizer used in each province and in China as a whole in 2018.

Province	Irrigated farmland area (ha)	Agricultural fertilizer application rate (kg/year)	Fertilizer usage (kg/ha/year)
Sichuan	2932.5 × 10^3^	235.2 × 10^7^	802.05
Gansu	1337.5 × 10^3^	83.2 × 10^7^	622.06
Qinghai	214 × 10^3^	8.3 × 10^7^	387.85
China	68,271.6 × 10^3^	5653.4 × 10^7^	828.07

### 4.2 Attitude

The perceptions of end-users are a key factor determining the degree of recycling of human excreta as fertilizer (Cofie et al., 2010). A section of the survey assessed existing attitudes of respondents to the concept of source separation of wastewater and reuse of human excreta by local residents.

#### 4.2.1 Attitude to reuse of human excreta as fertilizer

Our results showed that 72% (n = 297) of the interviewees were willing to produce human excreta-derived fertilizer and 70% (n = 289) were willing to promote its use in their community, while about 66% were willing to do both. Less than 5% of the interviewees (n = 20) stated that they would produce such fertilizer but were unwilling to promote its use. Multivariate logistic regression indicated that three factors, namely level of education, number of permanent household residents, and perceived social acceptability, significantly influenced the respondents' attitudes to human excreta (Table 5). However, only perceived social acceptability was strongly correlated and could explain the respondents' stated behavior (when social acceptability was excluded from the model, R^2^_McFadden_ decreased from 0.55 to 0.04) (Table 5).

**Table 5:** Results of multivariate logistic regression analysis on respondents' willingness to produce fertilizer derived from human excreta, and the strength of the association in the full multivariate model and in multivariate models excluding one variable at a time.

Variable	Multivariate model parameters			Strength of association	
	Odds ratio	*p*-Value		Model	R^2^_McFadden_
Province				Multivariate without ‘Province’	0.55
Gansu	Reference				
Qinghai	0.74	0.65			
Sichuan	4.29	0.15			
Gender (female)	1.43	0.46		Multivariate without ‘Gender’	0.55
Age				Multivariate without ‘Age’	0.56
≤30	Reference				
31– 40	1.55	0.62			
41– 50	0.51	0.47			
51– 60	0.66	0.64			
≥60	0.56	0.56			
Education				Multivariate without ‘Education’	0.53
None	Reference				
Primary school	5.88	**0.004**	***		
Junior high school	1.23	0.75			
High school	0.83	0.84			
College	0.09	**0.03**	**		
Bachelor degree or above	0.65	0.79			
Agriculture as occupation (yes)	1.22	0.69		Multivariate without ‘Agriculture as occupation’	0.55
Income (CNY)				Multivariate without ‘Income’	0.56
≤10,000 yuan	Reference				
10,000–20,000 yuan	1.02	0.97			
20,000–40,000 yuan	1.63	0.39			
40,000–60,000 yuan	1.69	0.60			
≥60,000 yuan	0.35	0.54			
Number of permanent household residents	0.75	**0.03**	**	Multivariate without ‘Number of permanent household residents’	0.54
System				Multivariate without ‘System’	0.55
Dry	Reference				
Flushing	0.44	0.38			
Current use of excreta in agriculture (yes)	2.20	0.15		Multivariate without ‘Current use of excreta in agriculture’	0.55
Is social acceptability important? (yes)	281.90	**<2e−16**	***	Multivariate without ‘Social acceptability’	0.04
				**Multivariate (all variables)**	**0.55**

Bold: it is significant.

*p < 0.1; ** p < 0.05; *** p < 0.01.

The number of permanent household residents was negatively correlated with willingness to produce excreta-derived fertilizers. This is because the smaller the number of permanent household residents, the smaller the amount of excreta produced (Table 5). Consequently, pits are emptied less frequently, so the work of handling and treatment is easier. Households with pit latrine-based sanitation were more willing to use excreta as fertilizer when the operation became easier. We also found that the lower the level of education, the higher the willingness to use human excreta (Table 5). Although the reasons for such stated behavior are difficult to decipher, we presumed that people with higher education tend to be more concerned about labor costs. Chemical fertilizers are inexpensive, easy to procure, and easy to apply (Savari and Gharechaee, 2020), while in the three provinces we surveyed the sanitation chain was in a poor state. As a result, applying excreta-derived fertilizer would increase food production costs and require more labor.

**Fig. 3:**
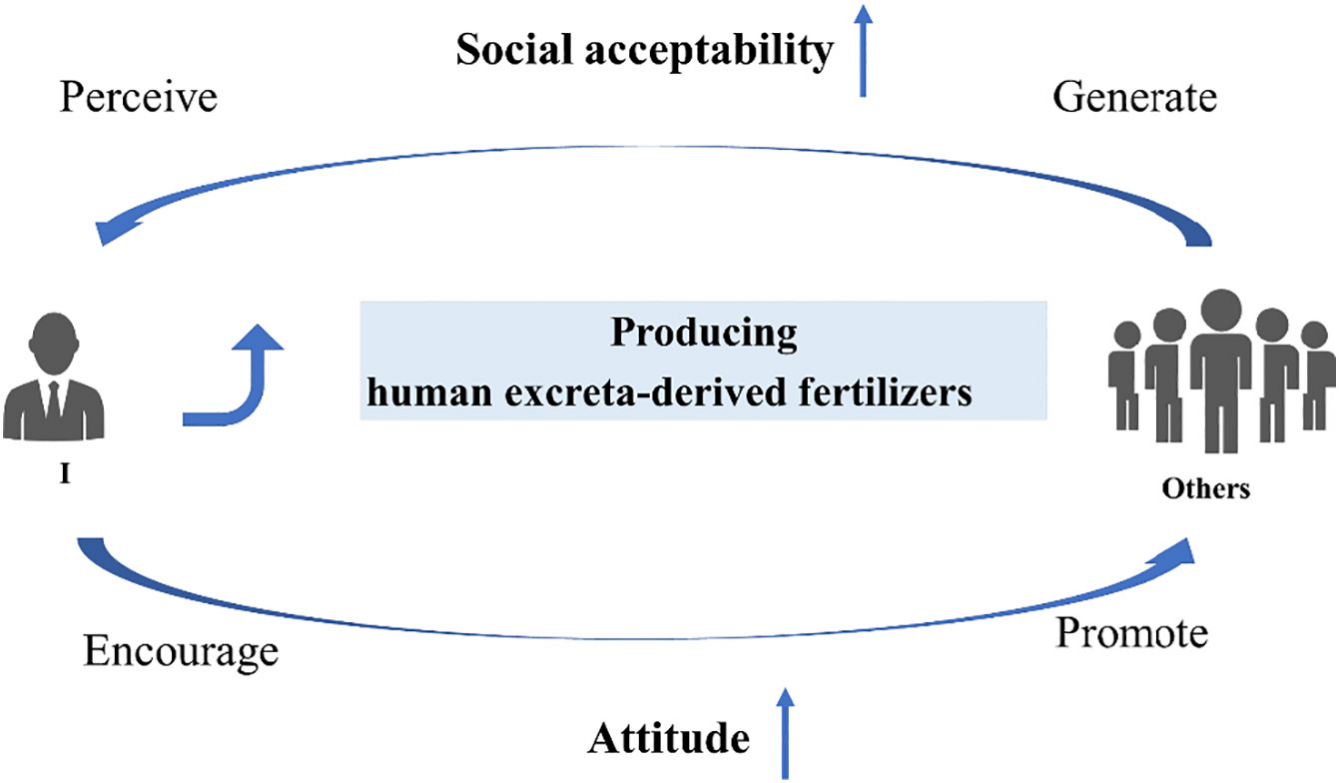
How perceived social acceptability affected the attitudes of respondents.

To explore the influence of social norms on household willingness to recycle excreta, we asked the respondents whether they would be open to promoting this practice. We explained that promotion not only means letting others use excreta as fertilizer, but also includes introducing, demonstrating, and teaching others how it can be carried out (Savari and Gharechaee, 2020). Almost 70% (n = 289) of the interviewees stated that they were willing to promote excreta recycling. Perceived social acceptability had the strongest association with respondents' attitude to use of human excreta among all the variables considered (excluding social acceptability from the multivariate model reduced R^2^_McFAdden_ from >0.5 to <0.05 in this case) (Table 5). In other words, perceived social acceptability was directly related to the interviewees' attitude, i.e., people are more likely to use human excreta in agriculture if they strongly perceive social acceptability of reusing human excreta as fertilizer. This can be crucial for propagating the practice in society (Fig. 3). Our findings are consistent with those in a previous study in Southern India, where farmers who knew someone using human urine as fertilizer never thought it was a bad idea to use it on their own crops (Simha et al., 2017). They are also in line with recent findings in a multinational survey, where social norms and cognitive awareness of the potential benefits and risks of urine reuse featured strongly in shaping respondents' attitudes to consuming urine-fertilized food (Simha et al., 2020). However, our results differed from those of a survey conducted at a university in Southern India (Simha et al., 2018), probably because of differences in the social environment. In rural China, people often have close social interactions so villagers often care about what others think of them, and a tendency for following the mainstream is very common. If many people have a positive attitude to human excreta-derived fertilizer, it can be expected that majority of villagers will adopt the same attitude, to be consistent with the collective. In China, crops grown with human excreta are traditionally considered safer and healthier than those grown with chemical fertilizer (Ferguson, 2014), and farmers view this practice as organic agriculture (Taghikhah et al., 2021).

#### 4.2.2 Barriers to reusing human excreta as fertilizer

**Fig. 4:**
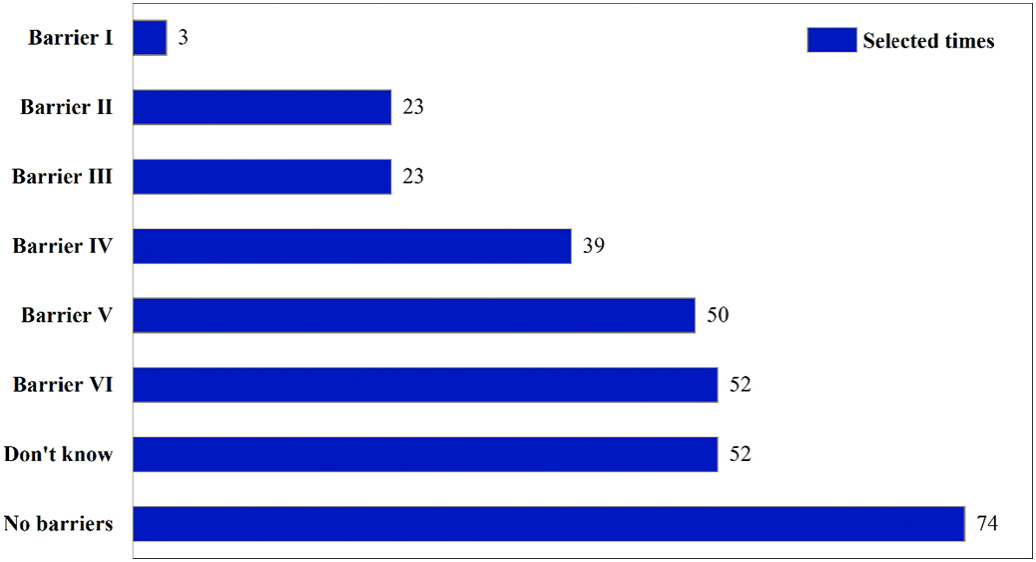
Perceived barriers to use of human excreta-derived fertilizer. Barrier I = small amount, no need for reuse; Barrier II = operator health problems; Barrier III = unknown quality of excrement fertilizer; Barrier IV = unbearable odor; Barrier V = cost; Barrier VI = insufficient technology.

A multiple-choice question, answered by 280 interviewees, asked about perceived barriers to implementation of excreta recycling. Overall, insufficient/inappropriate technology, high costs, and bad odors were most frequently cited as the major barriers to using human excreta as fertilizer (Fig. 4).

These findings are in line with the status of the sanitation chain in the three provinces surveyed. The majority of the households surveyed used a simple pit to collect and store excreta. In such toilets, bad odors are common both when the toilets are used and when they are emptied manually using shovels, buckets, or trolleys. Cost-related issues have also been mentioned in other studies (Zhang et al., 2020). For instance, labor costs for storage and transportation as a result of lack of machinery are a key barrier. In addition, many interviewees believed that using feces and urine without manual labor is “very expensive” under the current conditions. The most unexpected finding of our survey was that about half of the respondents perceived no barriers to use of excreta-derived fertilizers in agriculture, or were unaware of any barriers. This is consistent with previous findings that farmers applying manure may be unaware of any barriers to its use (Zhang et al., 2020). Overall, our results are similar to those found by others in the USA (Segrè Cohen et al., 2020), Europe (Lienert and Larsen, 2010), and India (Simha et al., 2017). High acceptance of excreta-derived fertilizers in our case could be linked to former agricultural traditions in China, where excreta use as fertilizer was common practice (Liu et al., 2014; Reganold and Wachter, 2016).

#### 4.2.3 Attitude to source separation

**Fig. 5:**
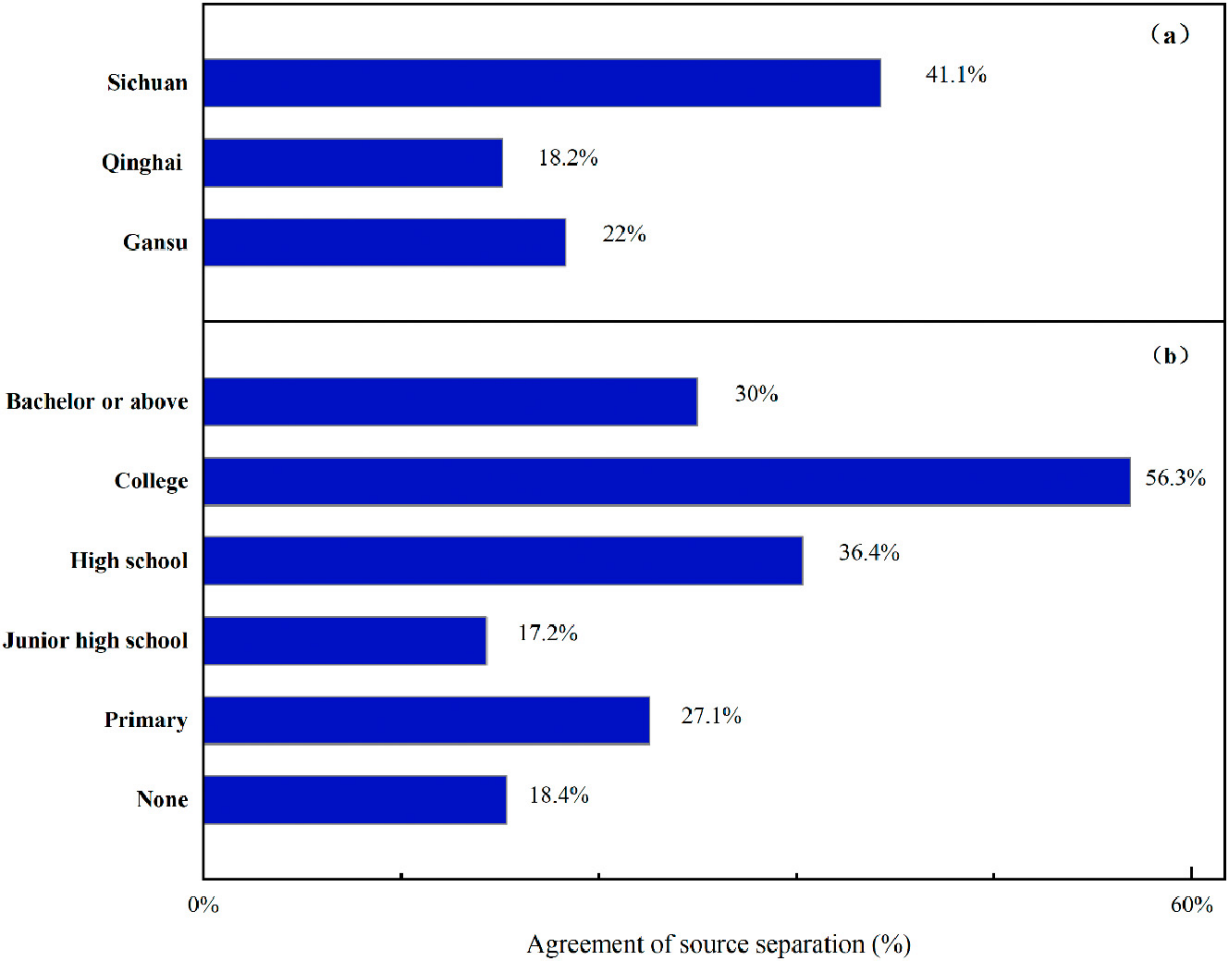
Acceptability of source separation of excreta (a) in the three provinces surveyed and (b) among respondents with different education levels.

Separating urine and feces at source can help efficiently recycle nutrients, but its practical implementation requires public acceptance (Ishii and Boyer, 2016). In our survey, only 24% of households agreed that these two fractions should be collected separately. Logistic regression showed that none of the variables included had any significant strength of association (*R*_*McFadden*_^2^ of the model with all variables was 0.06) (Supporting information, Table S1). More than 40% of households in Sichuan agreed that urine should be collected separately, twice the rate in the other two provinces (Fig. 5). In Sichuan, the Wenchuan earthquake of 2018 resulted in widespread loss of infrastructure, including traditional knowledge of toilets. The government provided biogas-linked toilets in the subsequent 12th Five-Year plan for the province, which also built awareness of these toilets. By contrast, Qinghai and Xining provinces have never experienced large-scale toilet reconstruction, which is perhaps why locals there were less receptive to new technologies. In the words of two of the respondents: “The situation in rural areas has always been like this, we cannot change it” and “I do not understand new things, and I do not want to try them”.

It has been reported that stakeholders in China presented with the concept of source separation of toilet wastes initially like and approve the concept, tend to be optimistic, and expect implementation of source-separating technologies to increase significantly within a relatively short time span (20 years) (Medilanski et al., 2016). So far, however, the level of the implementation of such technologies, especially in rural areas, has remained low. Moreover, social acceptance of the concept among end-users in China is lower than that reported for end-users in Switzerland (Pahl-Wostl et al., 2003), Sweden (Mcconville et al., 2017), India (Simha et al., 2018; Simha et al., 2017), and the USA (Lamichhane and Babcock, 2013). Further research is required to determine why this is the case.

## 5 Study implications

Overall, we identified many problems across the sanitation service chain in all three provinces surveyed. The simple pit latrine was the most common toilet in the survey villages and, although these toilets are cheap and easy to build, they may not provide sufficient protection against contamination from excreta. When pits are not lined or are dug in inadequate soil (i.e., rocky or coarse texture), leachate from toilets can transport viruses, bacteria, and nutrients (e.g., nitrates) from excreta to nearby groundwater or shallow water bodies. This poses a threat to human health if the water is used for human consumption (Sadler et al., 2016; Graham and Polizzotto, 2013).

We also found that pit latrines in the study area are managed as containers rather than as treatment processes for excreta (i.e., when a pit fills up, the owner empties it to continue using it, regardless of the sludge status), thereby generating incompletely treated excreta. This is openly disposed of in the environment, increasing the probability of human exposure. Most rural respondents opted for manually emptying the pits by themselves, which was the best handling option because of low cost and feasibility. This practice was carried out without protection, increasing the risk to workers' health, since aerosols formed during emptying operations can contain fecal bacteria and even respiratory viruses such as SARS (Xiao et al., 2020). Contact with high concentrations of parasites that are normally confined to pits is also very likely (Farling et al., 2019). Additionally, pit latrines can facilitate emergence and spread of antimicrobial resistance among fecal pathogens because of the long-term storage of fecal sludge (Beukes et al., 2017), thereby causing difficulty in treating infections. However, determining whether pit latrines were implemented under appropriate conditions was beyond the scope of this study, so the aforementioned risks remain speculative. Further investigation is required to quantitatively assess these risks, given the wide use of pit latrines found in this study and the magnitude of the rural population in China. The absence of suitable sanitation services may result in direct loss of the health gains achieved through elimination of open defecation (Balasubramanya et al., 2017; Schmidt, 2014). The lack of awareness of hygiene can impede improvement of pit latrine-based sanitation services, through what can be aptly described as D-D-D-O:

***Do not know what is right.*** The respondents were unaware of the MOHC guidelines and standards on hygienically disposing of/reusing human excreta and simply tended to follow what they felt was the appropriate way of doing things.
***Do not see alternatives.*** Most toilets in rural areas are manually self-emptied. Hiring suction trucks costs money and toilets usually are difficult for trucks to access, as the terrain tends to be rugged. Therefore, households are left with no option other than to empty the pit themselves, even though they may be reluctant to do this. Many interviewees echoed the sentiment “Such are the conditions in rural areas, and we are used to them.”
***Do not have a choice.*** Low/middle income rural areas, such as those we surveyed, invariably have relatively backward economies. The interviewees mentioned that they had few options for purchasing toilets, indicating that the product diversity of their local toilet market was low. In addition, economically underdeveloped areas are more likely to be unaware of the latest sanitation technologies and products. As a result, most of the respondents knew of little beyond flush toilets and simple pit latrines. As building flush toilets is difficult, they simply accepted their current situation.
***Over-reliant on government.*** Very few interviewees voluntarily improved their toilets. Many thought that the government should do everything related to sanitation. Although some people had ideas on sanitation improvement, they felt that they had no ownership or right to decide on such issues, probably because they are accustomed to the government intervening or because communication between policymakers and rural residents is lacking. As a consequence, respondents tend to think about what the government should do, instead of what they themselves can do.

In China, campaigns such as the Bill and Melinda Gates Foundation's “Reinvent the Toilet Challenge” (RTTC-China) and competitions such as “Technology Innovation in Building and Renovating Restrooms in the Country's Rural Areas”, initiated by the Ministry of Agriculture and Rural Affairs, have resulted in the development of many novel toilet designs and products for non-sewage toilet systems. However, in rural regions with poor economies or in areas prone to drought and cold climates, effective sanitation strategies remain underdeveloped. Moreover, very few people seem to have access to, or are aware of, new government policies that support improvement of rural sanitation. For example, only 2.9% of our survey respondents knew that the government has initiated a new Rural Toilet Revolution by making funds available for reconstruction and policies that promote the use of excreta as an organic fertilizer. This evident communication gap between policymakers and rural households needs to be addressed urgently.

## 6 Conclusions

In this study, we surveyed 414 villagers in rural regions across western China to assess the current status of the sanitation service chain and attitudes to human excreta management. Among several issues we discovered, poor hygiene was the main problem in use and management of pit latrine-based sanitation systems, which were used by 86.2% of households. Around 70% of the interviewees acknowledged that human excreta could be a valuable crop fertilizer and a similar proportion were willing to promote its use in local agriculture. Perceived social acceptability was the factor most strongly and positively associated with respondents' attitudes to production of human excreta-derived fertilizer. However, only 24% of respondents were positive to the concept of source separation of excreta. Technology demonstrations, guidance, and education could be effective ways to promote and develop source-separating sanitation systems in rural China and effectively support the country's ongoing Rural Toilet Revolution.

## CRediT authorship contribution statement

**Shaomin Guo:** Investigation, Methodology, Data curation, Writing – original draft. **Xiaoqin Zhou:** Conceptualization, Methodology, Writing – review & editing. **Prithvi Simha:** Writing – review & editing. **Luis Fernando Perez Mercado:** Software, Formal analysis. **Yaping Lv:** Writing – original draft. **Zifu Li:** Supervision, Writing – review & editing.

## Declaration of competing interest

The authors declare that they have no known competing financial interests or personal relationships that could have appeared to influence the work reported in this paper.

## Appendix A Supplementary data

Chinese rural residents’ attitude to the human excreta-derived fertilizer. Image 1
