# Supplementary material for: Poor awareness and attitudes to sanitation servicing can impede China's Rural Toilet Revolution: Evidence from Western China
Source: Sci Total Environ. 2021 Nov 10;794:148660. doi: 10.1016/j.scitotenv.2021.148660 (PMC8434418; doi:10.1016/j.scitotenv.2021.148660)
Supplement: Supplementary file 1 — Chinese rural residents’ attitude to the human excreta-derived fertilizer. [file mmc1.docx]

**Supplementary Information**

**Poor Awareness and attitudes to Sanitation Servicing can impede China’s Rural Toilet Revolution: evidence from Western China**

**Shaoming Guo^a,#^, Xiaoqin Zhou^a,#^ , Prithvi Simha^b,*^，Luis Fernando Perez Mercado^b,c^，Yaping Lv^a^, and Zifu Li^a,*^**

**^a^***Beijing Key Laboratory of Resource-oriented Treatment of Industrial Pollutants, School of Energy and Environmental Engineering, University of Science and Technology Beijing, Beijing 100083, PR China, Beijing PR China, 100083*

**^b^***Department of Energy and Technology, Swedish University of Agricultural Sciences, , Box 7032, SE-750 07 Uppsala, Sweden*

**^c^***Center for Water and Environmental Sanitation (Centro de Aguas y Saneamiento Ambiental, CASA), Universidad Mayor de San Simon, Calle Sucre y Parque Latorre, Cochabamba, Bolivia*

**^#^**These two authors made equal contributions. ^*^Corresponding author.

**Content:**

| Part I : | Questionnaire on the status of Chinese rural toilets |
| --- | --- |
| Part II : | Sample R code for logistic regression |
| Table S1 : | Results of multivariate logistic regression analysis on the acceptability of source-separating sanitation systems, and strength of association for the full multivariate model and for multivariate models excluding one variable at a time. |

**Part I. Questionnaire on the status of Chinese rural toilets**

Sites：Province: City: Country: Village: Hamlet:

Date: Interviewer：

**【Section A】**

Q1 Gender

1. Female B. Male

Q2 Age

1. ≤30
2. 31~40
3. 41~50
4. 51~60
5. ≥60

Q3 Education

1. None
2. Primary school
3. Junior high school
4. High school
5. College
6. Undergraduate college and above

Q4 Occupation

1. Unemployed
2. Farmer
3. Worker
4. Businessman
5. Students
6. Other

Q5 Family annual net income

1. ≤10 000 Yuan
2. 10 000-20 000 Yuan
3. 20 000-40 000 Yuan
4. 40 000-60 000 Yuan
5. ≥60 000 Yuan
6. Don’t know

Q6 Number of resident members in household

1. 1 B. 2 C. 3 D. 4 E. 5 F. 6 G. ≥7

**【Section B】**

Q7 Which kind of toilet do you have in your household currently？

1. Dry latrine
2. Flushing toilet
3. Public or shared toilet
4. Other types

Q8 How do you storage your family excreta？

1. Three-septic-tank type;
2. Double-vault funnel type;
3. Biogas-linked toilet;
4. Urine-feces division toilet;
5. Integrated flushing toilet;
6. Double pit alternate type;
7. Simple pit alternate
8. Public or shared toilet
9. Other toilets

Q9 How do you transfer your family’s excreta?

1. Transported by myself
2. Transported by sewage pipe
3. Transported by excrement suction truck
4. Don’t know

Note：If you use the excrement suction truck, please tell us the frequency of emptying and cost

Q10 What’s the final destination of your family’s excreta?

1. Store for a period of time after collection, then use it as crop fertilizer (storage time≥30 days)
2. Use it as crop fertilizer directly after collection
3. After collecting through the septic tank, transport it by the suction truck, but don’t know how the excrement disposed of after that
4. Collected in the sewer pipe, and then enters the sewage treatment plant
5. Seeps directly into the ground through the pit
6. Dumped in the environment
7. Don’t know

**【Section C】**

Q11 Would you like to produce human excreta-derived fertilizers?

1. Yes B. No

Q12 Would you promote the practice of producing human excreta-derived fertilizers? (Let other people do this?)

1. Yes B. No

Q13 What do you think are the barriers to reuse human excreta as organic fertilizer? (More than one answer can be chosen)

1. Little dosage, no need to reuse it
2. Operator health problems
3. The quality of excrement fertilizer
4. Unbearable odor
5. The cost
6. Insufficient technology
7. No barriers
8. Don't know

Q14 Do you think feces and urine should be separated?

1. Yes
2. No
3. Don’t know

Q15 How should the source-separated urine be handled? (More than one answer can be chosen)

1. Reuse as crop fertilizer
2. Treated by sewage system
3. Make biogas with livestock excrement
4. Used as water for lawn/flower
5. Used for power generation
6. Direct discharge into surface water sources (rivers, oceans)
7. Don't know

Q16 How should the source-separated feces be treated? (More than one answer can be chosen)

1. Reuse as crop fertilizer
2. Treated by sewage system
3. Make biogas with livestock excrement
4. The septic tank is treated and discharged into surface water sources (river, ocean)
5. Dilute and discharge into surface water source (river, ocean)
6. Dump to landfill for incineration
7. Don't know

Q17 Do you know the subsidy policy for organic fertilizer?

1. Yes B. No

Final question: Would you allow us to take some pictures of the toilet?

**Thank you for participating!**

**Part II. Sample R code for logistic regression**

# survey = data

### Variables:

# province

# gender

# age

# education

# occupation = Occupation as farmer or not

# income

# family.size = Number of family residents in the household

# system = Which kind of dry toilet do you have in your household?

# current.agriculture = Final destination of family’s excreta as crop fertilizer

# social.accept = Promoting the practice of producing fertilizers from human excreta?

# Q11.Produce = Would you like to produce fertilizers from human excreta?

### Logistic regression for Question 11 (Willingness to produce fertilizers from human excreta) as dependent variable with one single independent variable (gender)

produce<-glm(formula= Q11.Produce ~gender,

family = "binomial", data = survey, na.action=na.omit, control = glm.control(maxit=50)))

summary(produce)

**Table S1. Results of multivariate logistic regression analysis on the acceptability of source-separating sanitation systems, and strength of association for the full multivariate model and for multivariate models excluding one variable at a time**

| Variable | Multivariate model parameters | | | | Strength of association | |
| --- | --- | --- | --- | --- | --- | --- |
|  | | Odds Ratio | *p*-value |  | Model | R^2^_McFadden_ |
| Provinces | |  |  |  | Multivariate without ‘Province’ | 0.06 |
| *Gansu* | | Reference |  |  |  |  |
| *Qinghai* | | 0.84 | 0.69 |  |  |  |
| *Sichuan* | | 3.06 | 0.04 | ****** |  |  |
| Gender (Female) | | 1.05 | 0.83 |  | Multivariate without ‘Gender’ | 0.07 |
| Age | |  |  |  | Multivariate without ‘Age’ | 0.07 |
| ≤30 | | Reference |  |  |  |  |
| 31~40 | | 0.88 | 0.83 |  |  |  |
| 41~50 | | 1.14 | 0.81 |  |  |  |
| 51~60 | | 0.98 | 0.97 |  |  |  |
| ≥60 | | 0.80 | 0.71 |  |  |  |
| Education | |  |  |  | Multivariate without ‘Education’ | 0.04 |
| None | | Reference |  |  |  |  |
| Primary school | | 1.48 | 0.26 |  |  |  |
| Junior high school | | 0.94 | 0.88 |  |  |  |
| High school | | 2.43 | 0.05 |  |  |  |
| College | | 5.04 | **0.02** | ****** |  |  |
| Bachelor degree or above | | 1.76 | 0.52 |  |  |  |
| Agriculture as occupation (yes) | | 1.01 | 0.97 |  | Multivariate without ‘Agriculture as occupation’ | 0.07 |
| Income (CNY) | |  |  |  | Multivariate without ‘Income’ | 0.06 |
| ≤10 000 yuan | | Reference |  |  |  |  |
| 10 000-20 000 yuan | | 1.01 | 0.95 |  |  |  |
| 20 000-40 000 yuan | | 1.31 | 0.40 |  |  |  |
| 40 000-60 000 yuan | | 1.08 | 0.89 |  |  |  |
| ≥60 000 yuan | | 0.27 | 0.26 |  |  |  |
| Number of permanent household residents | | 0.93 | 0.34 |  | Multivariate without ‘Number of permanent household residents’ | 0.07 |
| System | |  |  |  | Multivariate without ‘System’ | 0.07 |
| Dry | | Reference |  |  |  |  |
| Flushing | | 0.67 | 0.50 |  |  |  |
| Current use of excreta in agriculture (yes) | | 1.73 | 0.12 |  | Multivariate without ‘Current use of excreta in agriculture’ | 0.06 |
|  | |  |  |  | **Multivariate (all variables)** | **0.07** |

**Statistically significant at p=0.05.
